# Physician Career Choice and Satisfaction: Empirical Studies of Practicing Physicians

**DOI:** 10.5195/jmla.2020.967

**Published:** 2020-07-01

**Authors:** Barbara M. Pope

**Affiliations:** 1 bpope@pittstate.edu, Periodicals/Reference Librarian, Leonard H. Axe Library, Pittsburg State University, Pittsburg, KS

*Physician Career Choice and Satisfaction: Empirical Studies of Practicing Physicians* summarizes the results of three studies that detail how and why physicians choose the types of practices where they work, physicians' personal characteristics, and their satisfaction with their work. The author of the book's preface, John R. Graham, notes that the University of New Mexico's medical school was established in 1961 and that there was debate among its teaching faculty on how to best teach medicine (p. iv). He adds that people learn best when they have a reason to learn rather than learning and regurgitating facts, as research from George Miller at the time showed. Therefore, the “shift toward motivation for learning emerged” (p. iv).

Gerald Otis's initial data collection about physicians' career choice and satisfaction began in 1968 and captured information on the medical school's first graduating class (p. iv). Otis's research was born out of concern about the “apparent development of increasingly cynical attitudes of medical students…during” medical school (p. 10–11). The research also includes a study of practicing physicians and a study of medical students doing summer externships. Data collection ended in the mid-1980s due to funding issues, but Otis resumed it later (p. 7).

The research looked at data about medical students and followed up with them as physicians years later. The data include the distribution of physicians across the various medical specialties, personality types, geography, income, level of patient contact, test scores, grades, and types of practice. Otis notes that certain types of characteristics—such as the kind of community the physician grew up in, family size growing up, or personality characteristics—may lead a physician to certain medical specialties (p. 53–61).

However, in the 1960s, the Student American Medical Association members believed that low recruitment to primary care was due to a specialized approach to patients in medical school and a lack of exposure to primary care (p. 135). Thus, the Medical Education and Community Orientation Program (MECO) was proposed in 1969 to allow preclinical students to get primary care experience through short rotations of up to ten weeks in community settings (p. 135). In addition, participation in summer externship programs, where students are placed with a physician preceptor, can significantly affect students' “medical attitudes and career dispositions”; however, Otis adds that success depends on both “program characteristics and student characteristics” (p. 156).

Otis notes that a general assumption in the longitudinal study is that the degree of satisfaction with a career choice depends on the degree that personal preferences align with aspects of a career that satisfies those preferences (p. 13). For example, various types of practices, such as partnerships or solo practices, differ from one another as well as the characteristics of physicians who do well in them, which can influence physicians' satisfaction, or lack thereof, with their work.

Quenk refers to the need to increase the desirability of working in Veterans Affairs (VA) hospitals, which are unattractive for a number of reasons, including low income; however, there are other issues at play, including administrative factors, so Quenk thinks it is unlikely that higher pay would make a difference (p. 122–123). Quenk adds that data show there is sometimes variation in satisfaction from one specialty area to another (p. 122). Otis suggests that a consortium or alliance of medical schools should work together to continuously research the area of physician career choice and satisfaction (p. 157).

The text is readable but would likely not interest readers outside the medical field, as it contains material that some readers would find difficult to slog through. This reviewer believes the book's intended audience is teaching faculty or administrators at medical schools who need to select students who are likely to be successful in medical school and be satisfied with their career choices. In addition, it may be useful to medical school students who are uncertain about what type of specialty or practice to select as well as those who are interested in attending medical school. The text does have some errors, such as inconsistent type size, line and word spacing errors, occasional spelling errors, and inconsistent paragraph justification. However, that does not outweigh the value of the information to faculty and administrators in medical education, as well as potential medical students. Therefore, including it in an academic library collection that supports potential medical school students is also a possibility. Therefore, this reviewer recommends this book, but only in these contexts.

